# Haemostasis in Thyroid Surgery: Collagen-Fibrinogen-Thrombin Patch versus Cellulose Gauze—Our Experience

**DOI:** 10.1155/2016/3058754

**Published:** 2016-02-25

**Authors:** Nicola Tartaglia, Alessandra Di Lascia, Vincenzo Lizzi, Pasquale Cianci, Alberto Fersini, Antonio Ambrosi, Vincenzo Neri

**Affiliations:** Department of Medical and Surgical Science, University of Foggia, Luigi Pinto Street 1, 71122 Foggia, Italy

## Abstract

*Purpose*. Postoperative hemorrhage is fortunately uncommon but potentially life-threatening complication of thyroid surgery that increases the postoperative morbidity and the hospital stay. In this study we compare the efficacy of collagen patch coated with human fibrinogen and human thrombin (CFTP) (group C) and oxidized regenerated cellulose gauze (group B) versus traditional hemostatic procedures (group A) in thyroid surgery.* Methods*. From January 2011 to December 2013, 226 were eligible for our prospective, nonrandomized, comparative study. Patients requiring a video-assisted thyroidectomy without drain, “near total,” or hemithyroidectomy were excluded. Other exclusion criteria were a diagnosis of malignancy, substernal goiter, disorders of hemostasis or coagulation, and Graves or hyperfunctioning thyroid diseases. Outcomes included duration of operation, drainage volume, and postoperative complications.* Results*. Our results show a significant reduction in drainage volume in group C in comparison with the other two groups. In group C there was no bleeding but the limited numbers do not make this result significant. There were no differences in terms of other complications, except for the incidence of seroma in group B.* Conclusion*. The use of CFTP reduces the drainage volume, potentially the bleeding complications, and the hospital stay. These findings confirm the efficacy of CFTP, encouraging its use in thyroid surgery.

## 1. Introduction

Thyroidectomy is one of the most commonly performed operative procedures in general surgery and it is the primary treatment modality in various thyroid pathologies [1].

Despite ongoing refinements in techniques and surgical excisions (MiVAT, endoscopic, robotic, ToVAT), innovations in surgical instruments, and neuromonitoring, postoperative hemorrhage remains a fortunately uncommon but potentially serious complication of thyroid surgery, occurring in 0.3% to 4.2% of cases [2].

The complex anatomy of the anterolateral neck region with multiple major vessels as well as the unique presence of larynx-tracheal conduit makes avoidance of intraoperative and postoperative hemorrhage a particular priority for the surgeon.

Intraoperative bleeding obscures important anatomical structures such as the laryngeal nerves and parathyroid glands and increases the risk of their lesions and the rate of morbidity.

On the other hand, postoperative hemorrhage may cause airway compression and respiratory distress, as a result of laryngopharyngeal edema secondary to the impairment of venous and lymphatic drainage [3].

This unfortunate event, potentially life-threatening, increases the postoperative morbidity and the hospital stay.

Hence, the need for safer and more effective hemostatic procedures in thyroid surgery is mandatory.

During surgical history, numerous technical advances have emerged in hemostasis, the suture ligatures, vessel ligating clips, electrocoagulation by mono- or bipolar instruments, and topical hemostatic agents, the use of which is mainly promoted in the last years in thyroid surgery as in other surgical disciplines.

We decided to use the hemostatic products that are currently available in our operating room, an equine collagen patch coated with human fibrinogen and human thrombin (CFTP) and oxidized regenerated cellulose gauze, following our favorable initial experience with these topical hemostatic agents in general surgery.

So we proceeded to design a prospective study to compare the efficacy of CFTP and cellulose gauze to traditional procedures in patients undergoing thyroid surgery, in terms of blood loss and the incidence of hemorrhagic events, always in relation to other complications.

## 2. Material and Methods

From January 2011 to December 2013, 293 consecutive patients were scheduled for thyroid surgery at our surgical unit, but only 226 were eligible for our prospective, nonrandomized, comparative study. Patients requiring a video-assisted thyroidectomy without drain, “near total,” or hemithyroidectomy were excluded. Other exclusion criteria were a diagnosis of malignancy with need for lymph node dissection, substernal goiter, and disorders of hemostasis or coagulation. To ensure homogeneity between the three groups we excluded also those affected by Graves or hyperfunctioning thyroid diseases.

Patients were divided into three groups, each employing a different means of hemostasis:

 
*group A* (69 patients) associated with the use of classic common surgical procedures of hemostasis (ligatures and electrocauterization);
 
*group B* (81 patients) with the use of oxidized regenerated cellulose gauze, which was placed in the thyroid cavities to cover the trachea and lateral spaces containing laryngeal nerves, parathyroid glands, and vessels legated during thyroidectomy;
 
*group C* (76 patients) with the application of CFTP that was cut into smaller pieces and placed over the areas at major risk, typically above perineural vessels.

All the patients underwent otorhinolaryngology control and ultrasound control preoperatively.

Patients were allocated to group arbitrarily by the surgical team at the beginning of each operation.

Because all patients were operated on by the same surgical team with great neck surgical experience, several key aspects of the procedures were standardized.

Informed consent was provided by all patients.

All three groups were comparable in terms of patient characteristics and thyroid weight (Table 1).

The mean age was 54 years and 74% were female.

The weight of the gland was detected immediately after his excision and even before it was included in paraffin. The weight was detected with the aid of a precision electronic balance.

All procedures were performed under general anesthesia.

Traditional thyroidectomy was carried out with hemostasis achieved by monopolar and bipolar coagulator and knot-tie technique. Recurrent laryngeal nerves and parathyroid glands were always identified.

Close to areas at risk from recurrent nerve or parathyroid damage, bipolar coagulation was applied only if ligation was inapplicable and unsafe for achieving hemostasis.

At the end of surgical procedures, a Valsalva maneuver was simulated to obtain an increase of the pulmonary pressure and subsequently of the systemic arterial pressure before wound closure for detection of bleeding vessels.

Only after this maneuver, topical hemostatic agents were applied in the patients of groups B and C with the primary aim to achieve hemostasis of this minor bleeding, if present, and the secondary one to prevent postoperative hematoma.

In addition to this, the application of these topical products can act as a barrier protecting the surgical area from the possible damage related to the use of a suction drainage.

In fact, before closing the wound, two small size (10 Ch) bilateral suction drain tubes were placed into thyroid cavities. These were removed 24 hours after surgery.

Outcomes measures included duration of operation, drainage volume during the first 24 hours as primary endpoint, and the occurrence of postoperative complications as secondary one.

After 24 hours, drainage volume, considered in this study, results from the sum of the volume of the contents drained by two drain tubes.

Measures of morbidity included incidence of hematoma, seroma, wound infection, transient hypoparathyroidism, and recurrent laryngeal nerve palsy.

Mean hospital stay was 3 days (range: 2–5) and follow-up for this study ended 1 month postoperatively.

### 2.1. Statistical Analysis

One-way ANOVA with Tukey's post hoc tests was performed for statistical comparison of the groups.

A *p* value of less than 0.05 was considered statistically significant.

## 3. Results

A summary of our results is reported in Table 2.

There were no significant group differences in terms of mean duration of surgery.

After 24 hours, drainage volume was significantly reduced in CFTP group in comparison with the other groups (*p* < 0.001); instead no differences were found between traditional group and cellulose gauze group (*p* = 0.740).

The secondary endpoint of the study was the rate of postoperative complications.

Three patients (3/226, 1.3%) in the general population had postoperative bleeding, requiring wound exploration under anesthesia: 2 in group A (2.9%) and 1 patient in group B (1.2%), without significant differences between the three groups. No cases of postoperative hematoma occurred in the CFTP group. In our study, postoperative cervical hematoma occurred within 6 hours after the surgery. All of these patients recovered well after the reexploration, during which obvious bleeding site was detected, from the surface of the strap muscles in one case and from vessels near the end of the recurrent laryngeal nerve in the others.

Seroma, appearing usually 4–6 days after surgery, was also significantly different between groups (*p* = 0.010) because we found only 5 cases in group B.

In this study no case of wound infection was observed and no significant differences were obtained in the occurrence of transient postoperative hypoparathyroidism and transient recurrent laryngeal nerve palsy (*p* > 0.05).

There were no statistical differences in terms of postoperative morbidity among the 3 groups as shown in Table 2. Total morbidity was 20.3% in the traditional group, 18.5% in the cellulose gauze, and 14.5% in the CFTP group (all comparison, *p* > 0.05).

## 4. Discussion

Thyroidectomy and neck dissection were the most commonly performed surgeries on the neck and had low morbidity rates. Hematoma formation after thyroidectomy is a well-known complication, but with improved surgical technique and meticulous hemostasis, it has become a rare occurrence [4]. Nevertheless, if not detected early and managed properly, it is potentially life-threatening.

In fact, patients with postoperative cervical hematoma required reoperation and longer hospital monitoring.

The patient shows respiratory distress and pain or pressure sensation in the neck.

Progressive neck swelling, suture line bleeding, and stridor are the most common signs in association with significant drain tube losses if a drain has been left in situ.

In 89% of cases postoperative hematoma occurs within 12 hours after surgery and 72% within 6 hours [5].

Risk factors associated with postoperative hemorrhage are classified into those related to the patient and those related to thyroid diseases and to surgical technique.

Evidently, patients with coagulation disorders and chronic renal failure or patients that take antiaggregant and/or anticoagulant medications present a higher risk of bleeding [6].

Other risk factors are represented by Basedow's disease, toxic multinodular glands because of the increased vascularity of the thyroid, and intrathoracic goiters because of the altered vascularity and the increased size of the gland and the greater extension of the operating field.

Surgical technique evidently plays a crucial role in preventing postoperative bleeding: firstly the mode of access and the dissection of strap muscles, the means of hemostasis, and the presence of residual thyroid tissue.

The use of instruments as the harmonic scalpel and radiofrequency or thermal devices also improves vessel sealing and several studies confirm their effectiveness to prevent hemorrhagic events [7–9].

Also anesthetic factors play a crucial role in prevention of neck hematoma such as a smooth extubation without significant coughing and the control of both postoperative vomiting and pain to avoid raised venous and arterial pressures.

The ability of suction drainage to reduce the incidence of postthyroidectomy hemorrhage is a debated topic. The aim of drain tube in thyroid surgery is to obliterate the dead space, to evacuate collected blood and serum, and to early detect postoperative bleeding. Recently, the use of drains has decreased considerably and several reports regarding the function of drains in thyroid surgery have not justified their use [10, 11] and have demonstrated their association with surgical site infections and prolonged hospital stay [12].

Means to prevent and control intra- or postoperative bleeding have always been a topic of most importance. The first step in preventing postoperative hemorrhagic complications is meticulous hemostasis during surgical procedures.

During the last few years various topical hemostatic agents have become available to improve hemostasis during surgery.

The first data in literature date back to 1987, when Auvinen et al. [13] examined the effect of the antifibrinolytic drug tranexamic acid on perioperative bleeding. Consequently, Lachachi et al. [14] used fibrin sealant as hemostatic local agent. In 2006, Tonante et al. [15] stated the use of Floseal, matrix hemostatic agents, like other studies [16, 17]. Amit et al. [18] examined in 2013 the effectiveness of Surgicel, an oxidized cellulose patch.

Products we use in our center include oxidized cellulose gauze and CFTP, both of which are widely used.

After preliminary successful experience with these hemostatic agents in general surgery, we initiated our study to verify their effectiveness in thyroid surgery and to compare them with conventional surgical hemostatic procedures.

The oxidized regenerated cellulose gauze acts initially as a mechanical barrier for blood and subsequently becomes a viscous mass that is like an artificial clot. Hence, this product has no intrinsic hemostatic effect but instead induces hemostasis by providing a strong matrix for platelet adhesion and aggregation. This accelerates the formation of a platelet plug and acts as a catalyst in forming a fibrin clot. Its low pH causes localized vasoconstriction, further enhancing the hemostatic effect and minimizing the risk of infection. Then this is degraded and absorbed within a maximum period of 7 days.

CFTP is a sealant patch of equine collagen (support) medicated with thrombin and fibrinogen. When it is in contact with the blood, the fibrinogen and the thrombin are activated, giving rise to the final stage of the coagulation cascade, forming a fibrin network. The sponge firmly adheres to the tissue, and it will be absorbed completely within 12 weeks.

Our findings show no differences in terms of mean duration of surgery and total postoperative morbidity.

The use of oxidized cellulose gauze is associated with the relevant possibility of foreign body reactions that can cause undesirable effects, such as an increase in the incidence of seroma, as observed in this study with an incidence rate of 6.2%. No case was observed in the other 2 groups.

In this study, no hemorrhagic complications occurred in patients treated with CFTP, only one case in group B and two cases in the group of standard treatment.

Even if no statistical differences were found between the three groups, we obtained interesting data about the different bleeding sites. In fact, the only case of postoperative hematoma in group B (cellulose gauze) was due to bleeding of surface of the strap muscles; instead, the two cases of group A (standard treatment) were associated with bleeding from perineural vessels.

Postoperative hematoma has a multifactorial etiopathogenesis including slipping of ligatures and reopening of previously cauterized vessels, above all of the perineural vessels. This area represents a challenge for the surgeon due to hemostasis because of the nearness of the recurrent nerve. For this reason, Tonante et al. [15] conducted a study with another topical product for the control of bleeding at this site.

Our study put into evidence how the application of the topical hemostatic agents in the thyroid cavities, over this area at major risk, could prevent a postoperative hematoma due to its bleeding.

The reason may be related to the immediate efficacy of the topical hemostatic agent with respect to conventional surgical maneuvers performed to obtain bleeding control.

Nevertheless, the limited occurrence of hemorrhagic events of this study cannot confirm the efficacy of topical hemostatic agents to prevent hemorrhagic postoperative complications.

Our results show a significant reduction in drainage volume in the CFTP group in comparison with the other two groups. Even if the drains were removed after 24 hours from surgery in all patients because of the modest amounts, the reduction in drainage volume in the CFTP group led to discharge of these patients on POD (postoperative day) 2 instead of POD 3 as the other patients.

The additional cost associated with CFTP use was offset by a shorter postoperative stay.

## 5. Conclusions

Although uncommon, postoperative bleeding after thyroid surgery remains a potentially life-threatening complication. Whilst the surgical haemostatic armamentarium has progressed significantly in the last century, a surgical technique based on experience is essential.

The use of CFTP showed a statistically significant reduction in drainage volume. In the CFTP group there was no bleeding, unlike the other two groups, but the limited numbers do not make this difference significant. So we can say that the use of CFTP reduces the drainage volume; it potentially reduces bleeding complications; patients enjoy more comfort, by the shorter hospital stay and the faster recovery of normal daily activities. These findings confirm the efficacy of CFTP, promoting its use in thyroid surgery.

## Figures and Tables

**Table 1:** Patients baseline characteristics.

	A: standard treatment (69)	B: cellulose gauze (81)	C: CFTP (76)	Overall (226)	One-way ANOVA *p* value
Gender (M/F)	16/53	23/58	19/57	58/168	0.759
Mean age, years (range)	56 (18–82)	50 (19–79)	53 (20–75)	54 (18–82)	0.105
Mean ± SD thyroid weight (grams)	86.12 ± 10.2	88.34 ± 9.8	90.25 ± 10.5	88.76 ± 10.11	0.052

**Table 2:** Postoperative outcomes.

	A: standard treatment (69)	B: cellulose gauze (81)	C: CFTP (76)	One-way ANOVA *p* value	Post hoc Tukey test *p* value
Operation length, mean ± SD, (range) min	112 ± 26.63 (75–180)	111 ± 14.91 (90–140)	114 ± 30.62 (80–190)	0.679	A : B 0.899 A : C 0.758 B : C 0.672

Drainage volume, mean ± SD, (range) mL	80 ± 9.83 (70–100)	79 ± 12.19 (65–105)	60 ± 9.38 (50–80)	**<0.001**	A : B 0.740 **A : C p < 0.001** **B : C p < 0.001**

Postoperative hemorrhage, *n* (%)	2 (2.9%)	1 (1.2%)	0	0.315	A : B 0.637 A : C 0.283 B : C 0.758

Seroma, *n* (%)	0	5 (6.2%)	0	**0.010**	**A : B 0.026** A : C 0.899 **B : C 0.022**

Transient postoperative hypoparathyroidism, *n* (%)	8 (11.6%)	6 (7.4%)	7 (9.2%)	0.681	A : B 0.643 A : C 0.863 B : C 0.899

Transient recurrent laryngeal nerve palsy, *n* (%)	4 (5.8%)	3 (3.7%)	4 (5.3%)	0.824	A : B 0.806 A : C 0.899 B : C 0.886

Total postoperative morbidity *n* (%)	14 (20.3%)	15 (18.5%)	11 (14.5%)	0.641	A : B 0.899 A : C 0.622 B : C 0.766
